# *Saccharomyces boulardii* CNCM I-745 Supernatant Improves Markers of Gut Barrier Function and Inflammatory Response in Small Intestinal Organoids

**DOI:** 10.3390/ph18081167

**Published:** 2025-08-06

**Authors:** Louisa Filipe Rosa, Steffen Gonda, Nadine Roese, Stephan C. Bischoff

**Affiliations:** 1 Institute of Nutritional Medicine, University of Hohenheim, Fruwirthstr. 12, 70599 Stuttgart, Germany; louisa.homberg@uni-hohenheim.de; 2 MEDICE Arzneimittel Pütter GmbH & Co., KG, Kuhloweg 37, 58638 Iserlohn, Germany; s.gonda@medice.de (S.G.); n.roese@medice.de (N.R.)

**Keywords:** *Saccharomyces boulardii* CNCM I-745, diarrhea, gastroenteritis, inflammation, gastrointestinal barrier, antimicrobial peptides, tight junctions, mucosal protection, murine organoids

## Abstract

**Objectives**: *Saccharomyces boulardii* CNCM I-745, a probiotic yeast, is effectively used for the treatment of acute diarrhea as well as for the prevention and treatment of traveller‘s diarrhea and diarrhea under tube feeding. The underlying mechanisms are not fully elucidated. Both antitoxic and regulatory effects on the intestinal barrier, mediated either by the yeast or yeast-derived substrates, have been discussed. **Methods**: To examine the effects of *Saccharomyces boulardii* released substrates (S.b.S) on gastrointestinal (GI) barrier function, a murine small intestinal organoid cell model under stress was used. Stress was induced by lipopolysaccharide (LPS) exposure or withdrawal of growth factors from cell culture medium (GF_Red_). Stressed organoids were treated with S.b.S (200 µg/mL), and markers of GI barrier and inflammatory response were assessed. **Results**: GF_Red_-induced stress was characterized by disturbances in selected tight junction (TJ) (*p* < 0.05), adherent junction (AJ) (*p* < 0.001), and mucin (*Muc*) formation (*p* < 0.01), measured by gene expressions, whereby additional S.b.S treatment was found to reverse these effects by increasing *Muc2* (from 0.22 to 0.97-fold change, *p* < 0.05), Occludin (*Ocln*) (from 0.37 to 3.5-fold change, *p* < 0.0001), and Claudin (*Cldn*)7 expression (from 0.13 ± 0.066-fold change, *p* < 0.05) and by decreasing *Muc1*, *Cldn2*, *Cldn5*, and junctional adhesion molecule A (*JAM-A*) expression (all *p* < 0.01). Further, S.b.S normalized expression of nucleotide binding oligomerization domain (*Nod*)2- (from 44.5 to 0.51, *p* < 0.0001) and matrix metalloproteinase (*Mmp*)7-dependent activation (from 28.3 to 0.02875 ± 0.0044 ** *p* < 0.01) of antimicrobial peptide defense and reduced the expression of several inflammatory markers, such as myeloid differentiation primary response 88 (*Myd88*) (*p* < 0.01), tumor necrosis factor α (*Tnfα*) (*p* < 0.01), interleukin (*IL*)-6 (*p* < 0.01), and *IL-1β* (*p* < 0.001). **Conclusions**: Our data provide new insights into the molecular mechanisms by which *Saccharomyces boulardii* CNCM I-745-derived secretome attenuates inflammatory responses and restores GI barrier function in small intestinal organoids.

## 1. Introduction

*Saccharomyces boulardii* (*S. boulardii*) CNCM I-745^®^ was the first probiotic yeast developed in 1961 and approved for the prevention and treatment of diarrhea in both adults and children, including *Clostridioides difficile* (*C. difficile*) associated diarrhea [1,2,3,4]. *S. boulardii* CNCM I-745^®^ exerts direct therapeutic effects on bacterial toxins and pathogens and modulates the host’s intestinal barrier by improving immune response against infections [5,6,7,8,9,10]. Accordingly, it was demonstrated that a <3 kDa fraction of *S. boulardii* CNCM I-745 culture supernatant reduced LPS-mediated induction of co-stimulatory CD40 and CD80 molecules and decreased proinflammatory cytokine secretion [8]. In addition, *S. boulardii* has been associated with increased intestinal immunoglobulin A secretion [11], as well as with antitoxic and antimicrobial effects, including secreted proteases that directly degrade *C. difficile* toxins A and B [12,13]. Further, there is evidence that phosphatases, released by *S. boulardii*, exhibited the potential to deactivate *Escherichia coli* (*E. coli*) endotoxins [6].

It has been hypothesized that the clinical efficacy of *S. boulardii* CNCM I-745 against infection depends on complementary effects on the GI barrier. An intact intestinal barrier is essential for preservation of GI homeostasis and has become an important aspect of preventing and treating various diseases [14]. Disturbances of the intestinal barrier lead to increased intestinal permeability, promoting systemic inflammatory reactions and development of GI diseases such as inflammatory bowel diseases (IBD), irritable bowel syndrome, and viral or bacterial infections, as well as extraintestinal diseases such as metabolic dysfunction-associated fatty liver disease or type 2 diabetes [15,16,17,18,19]. Thereby, several factors such as genetic variations, microbial dysbiosis, chronic inflammation, but also disturbed TJ formation altered intestinal permeability [20,21,22,23,24]. There is evidence that inflammatory and immune-mediated mechanisms regulate gene expression within epithelial tissues, thereby affecting the structural and functional integrity of the GI barrier [25]. Mucins and tight junction proteins are important components of the intestinal epithelial barrier, and gene expression levels serve as reliable biomarkers for assessing disturbances in barrier function induced by microbial and inflammatory stimuli. Therefore, quantification of these GI barrier markers provides a mechanistic insight into the impact of inflammation on intestinal barrier function [20,26].

Furthermore, antimicrobial peptides (AMPs) play a key role for GI barrier function and pathogen defense [27]. In the small intestine, AMP formation occurs in specialized Paneth cells, which secrete several AMPs, including high amounts of α-defensins [28]. To provide antimicrobial activity, proteolytic activation dependent on the enzyme MMP7, which is colocalized in Paneth cell granules, is required [29,30]. Additionally, their antimicrobial activity against harmful bacteria, AMPs also act as a second line of defense by restricting bacterial translocation. Therefore, impaired Paneth cell antimicrobial peptide defense has been associated with increased bacterial translocation [20,31,32].

*S. boulardii* has been associated with increased TJ integrity between epithelial cells, decreased crypt hyperplasia, and reduced cell damage in *Citrobacter rodentium*-infected mice [33,34]. Furthermore, Crohn’s disease (CD) patients exhibited improved intestinal permeability when receiving a daily *S. boulardii* formulation [35]. Although *S. boulardii* CNCM I-745^®^ has been widely used for the treatment and prevention of gastrointestinal disorders, including antibiotic-associated diarrhea, the molecular mechanisms by which *S. boulardii* and its released components regulate GI barrier function during inflammatory processes are still poorly understood. We proposed that S.b.S modulates regulators of intestinal inflammation and epithelial barrier markers in murine organoids under stress conditions. Therefore, the present study investigated the effects of *S. boulardii* CNCM I-745^®^ supernatants on GI barrier function and inflammatory responses during stress by using an in vitro murine small intestinal organoid model. We hypothesized that S.b.S would modulate inflammatory markers and junctional proteins associated with mucosal barrier function.

## 2. Results

### 2.1. Determination of S.b.S Concentration 

**Figure 1:**
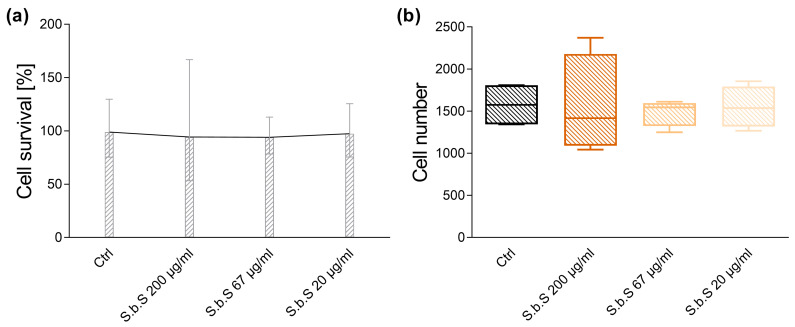
S.b.S does not modulate cell viability and cell number of small intestinal organoids. For MTT assay, murine small intestinal organoids were treated with S.b.S (200 µg/mL, or 67 µg/mL, or 20 µg/mL) or with RPMI as control for 30 h at 37 °C. Cell viability was assessed by MTT assay. Organoids were incubated with 7 µL MTT (500 mg/mL, 1 h, 37 °C, 5% CO_2_), followed by SDS-mediated Matrigel solubilization and DMSO extraction. Absorbance was measured at 562 nm. Cell survival in % (**a**) and total cell number (**b**) are shown (n = 4). Data are presented as means ± standard error of the mean (SEM) and were analyzed by Kruskal–Wallis test with Dunn’s multiple comparisons test. Differences between two groups were analyzed by using Mann–Whitney test (**a**,**b**).

Determination of S.b.S protein levels by BCA protein assay revealed protein concentrations in the expected range (2.95 mg/mL to 3.08 mg/mL, Figure S1). To exclude toxicological effects on organoids by S.b.S, a 3-[4,5-dimethylthiazol-2-yl]-2,5 diphenyl tetrazolium bromide (MTT) assay was performed. Exposure to S.b.S (200 µg/mL, 67 µg/mL, 20 µg/mL) had no significant effects on cell viability or cell number (Figure 1a,b, Tables S1 and S2).

**Figure 2:**
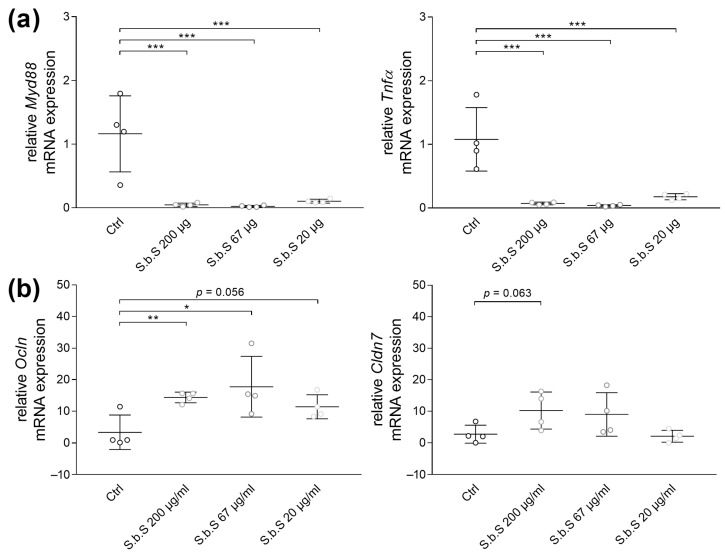
S.b.S exposition decreases inflammatory markers and induces TJ protein expression. Murine small intestinal organoids were treated with S.b.S (200 µg/mL, 67 µg/mL, 20 µg/mL) or RPMI as control for 30 h at 37 °C. Gene expression of *Tnfα*, *Myd88*, *Cldn7*, and *Ocln* was assessed by RT-PCR using the ΔΔCt method normalized to β-actin. Relative mRNA expression levels of *Myd88*, *Tnfα* (**a**), *Ocln*, and *Cldn7* (**b**) determined by quantitative RT-PCR are shown. Data are presented as means ± SEM (n = 4). Statistical analysis was performed by one-way ANOVA with Dunnett’s multiple comparisons test or Kruskal–Wallis test with Dunn’s multiple comparisons test. Differences between two groups were analyzed by using unpaired *t*-test or Mann–Whitney test. Significant differences to RPMI control are indicated as * *p*-value < 0.05; ** *p*-value < 0.01; *** *p*-value < 0.001. Statistical trends were defined as 0.05 < *p*-value < 0.1.

Examination of inflammatory markers showed that S.b.S at 200 µg/mL, 67 µg/mL, or 20 µg/mL decreased *Myd88* and *Tnfα* gene expression (*p* < 0.001, Figure 2a). Additionally, PCR analyses revealed that S.b.S at 200 µg/mL induced TJ gene expression of *Ocln* (*p* < 0.01) and *Cldn7* by trend (*p* = 0.0624), whereas lower S.b.S concentrations induced *Ocln* mRNA expression by trend (67 µg/mL, *p* < 0.05; 20 µg/mL, *p* = 0.056, Figure 2b). These results suggested that S.b.S is appropriate for use in organoid cell culture and improved inflammatory response and GI barrier function, especially at 200 µg/mL. Based on these findings, a concentration of 200 µg/mL S.b.S was defined for further experiments.

### 2.2. S.b.S Improves GF_Red_- and LPS-Dependent Disturbances of GI Barrier Function

#### 2.2.1. S.b.S Exposition Regulates Stress-Induced Changes in TJ and Muc Transcripts Expression

**Figure 3:**
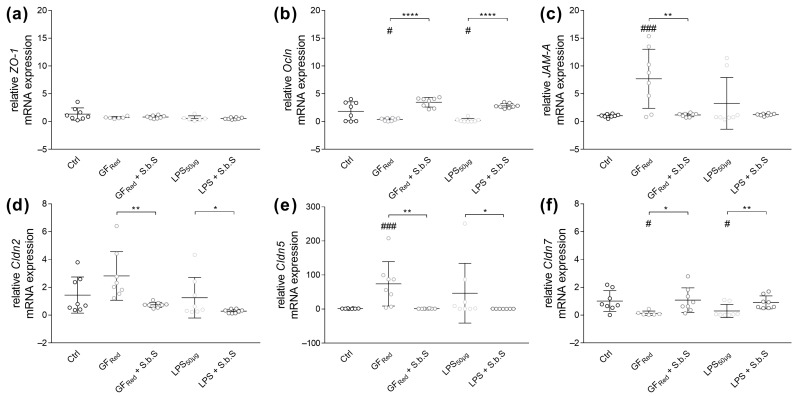
GF_Red_ and LPS disturb TJ and AJ proteins, which are normalized by S.b.S exposition. Murine small intestinal organoids were treated with S.b.S (200 µg/mL), or GF_Red_ ± S.b.S (200 µg/mL), or LPS (50 µg/mL) ± S.b.S (200 µg/mL), or RPMI as control for 30 h at 37 °C. Gene expression of *ZO-1*, *Ocln*, *JAM-A*, *Cldn2*, *Cldn5*, and *Cldn7* was assessed by RT-PCR using the ΔΔCt method normalized to β-actin. Relative mRNA expression levels of *ZO-1* (**a**), *Ocln* (**b**), *JAM-A* (**c**), *Cldn2* (**d**), *Cldn5* (**e**), and *Cldn7* (**f**) determined by quantitative RT-PCR are shown. Data are presented as means ± SEM (n = 8). Statistical analysis was performed by one-way ANOVA with Dunnett’s multiple comparisons test (**a**,**b**) or Kruskal–Wallis test with Dunn’s multiple comparisons test (**c**–**f**). Differences between two groups were analyzed by using unpaired *t*-test (**a**,**b**) or Mann–Whitney test (**c**–**f**). Significant differences to RPMI control are indicated as ^#^ *p*-value < 0.05; ^###^ *p*-value < 0.001. Significant differences between two groups are indicated as * *p*-value < 0.05; ** *p*-value < 0.01; **** *p*-value < 0.0001.

Gene expression analysis revealed that neither stress induction with modified CCM (reduced growth factors, GF_Red_) nor LPS had any effects on *ZO-1* mRNA expression (Figure 3a, Table S3). However, incubation with GF_Red_ as well as with LPS resulted in a decrease in *Ocln* mRNA expression (*p* < 0.05), whereas additional exposure to S.b.S induced *Ocln* mRNA expression (*p* < 0.0001, Figure 3b). The adherent junction (AJ) *JAM-A* exhibited special characteristics of being induced during inflammatory processes [36]. Similarly, stress induction by GF_Red_ induced *JAM-A* gene expression (*p* < 0.001), whereby these effects were absent when organoids were additionally treated with S.b.S (*p* < 0.01, Figure 3c and Figure S2). Furthermore, exposure of organoids with GF_Red_ increased *Cldn5* expression (*p* < 0.001), whereas *Cldn7* mRNA expression was reduced (*p* < 0.05, Figure 3d,e and Figure S2). Moreover, LPS treatment decreased *Cldn7* mRNA expression (*p* < 0.05, Figure 3e). Interestingly, additive incubation with S.b.S restored *Cldn2* (*p* < 0.01), *Cldn5* (*p* < 0.01), and *Cldn7* (*p* < 0.05) gene expression in GF_Red_-treated organoids, as well as *Cldn7* mRNA expression (*p* < 0.01) in LPS-stimulated organoids (Figure 3d,e and Figure S2).

**Figure 4:**
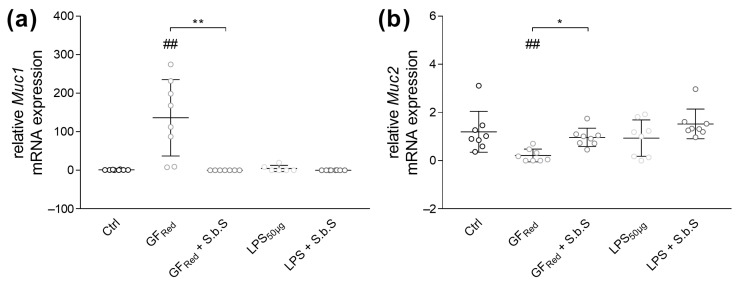
S.b.S at 200 µg/mL improves GF_Red_-induced disturbances in mucus formation. Murine small intestinal organoids were treated with S.b.S (200 µg/mL), or GF_Red_ ± S.b.S (200 µg/mL), or LPS (50 µg/mL) ± S.b.S (200 µg/mL), or RPMI as control for 30 h at 37 °C. Gene expression of *Muc1* and *Muc2* was assessed by RT-PCR using the ΔΔCt method normalized to β-actin. Relative mRNA expression levels of *Muc1* (**a**), and *Muc2* (**b**) determined by quantitative RT-PCR are shown. Data are presented as means ± SEM (n = 8). Statistical analysis was performed by Kruskal–Wallis test with Dunn’s multiple comparisons test (**a**,**b**). Differences between two groups were analyzed by using unpaired Mann–Whitney test (**a**,**b**). Significant differences to RPMI control are indicated as ^##^ *p*-value < 0.01. Significant differences between two groups are indicated as * *p*-value < 0.05; ** *p*-value < 0.01.

Further, stress induction by GF_Red_ was associated with an increased in *Muc1* (*p* < 0.01) and a decreased in *Muc2* mRNA expression (*p* < 0.01, Figure 4a,b, Table S3). Conversely, LPS exposure revealed no effects on *Muc1* and *Muc2* expression (Figure 4a,b and Figure S2). Additional incubation of GF_Red_-treated organoids with S.b.S was found to normalize *Muc1* and *2* gene expression, which was associated with a decrease in *Muc1* (*p* < 0.01) and an increase in *Muc2* (*p* < 0.05) gene expression (Figure 4a,b and Figure S2).

#### 2.2.2. S.b.S Normalizes Antimicrobial Peptide Defense in GF_Red_- and LPS-Treated Organoids

**Figure 5:**
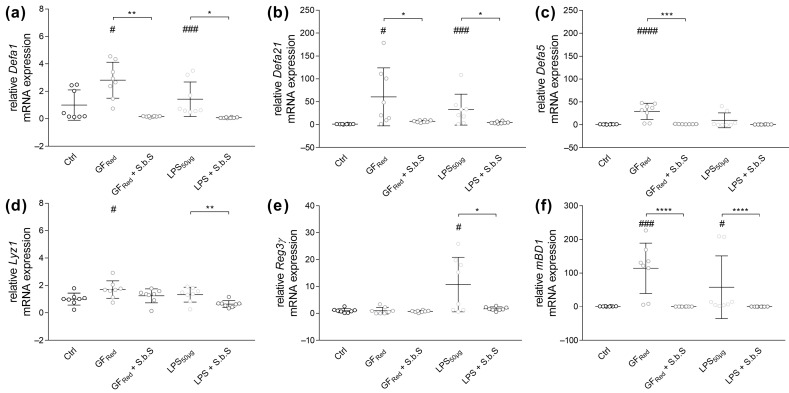
GF_Red_ and LPS activate antimicrobial peptide defense, which is normalized by simultaneous treatment with S.b.S at 200 µg/mL. Murine small intestinal organoids were treated with S.b.S (200 µg/mL), or GF_Red_ ± S.b.S (200 µg/mL), or LPS (50 µg/mL) ± S.b.S (200 µg/mL), or RPMI as control for 30 h at 37 °C. Gene expression of *Defa1*, *Defa21*, *Defa5*, *Lyz1*, *Reg3γ*, and *mBD1* was quantified by comparison to standard curves from serially diluted plasmid standards and normalized to β-actin copy numbers. Relative mRNA expression levels of *Defa1* (**a**), *Defa21* (**b**), *Defa5* (**c**), *Lyz1* (**d**), *Reg3γ* (**e**), and *mBD1* (**f**) determined by quantitative RT-PCR are shown. Data are presented as means ± SEM (n = 8). Statistical analysis was performed by one-way ANOVA with Dunnett’s multiple comparisons test or Kruskal–Wallis test with Dunn’s multiple comparisons test. Differences between two groups were analyzed by using unpaired *t*-test or Mann–Whitney test. Significant differences to RPMI control are indicated as ^#^ *p*-value < 0.05; ^###^ *p*-value < 0.001; ^####^ *p*-value < 0.0001. Significant differences between two groups are indicated as * *p*-value < 0.05; ** *p*-value < 0.01; *** *p*-value < 0.001; **** *p*-value < 0.0001.

In the present study we were able to show that stress induction in organoids by GF_Red_ increased α-defensin (*Defa*) 1 (*p* < 0.05), *Defa21* (*p* < 0.05), and *Defa5* (*p* < 0.0001) gene expression (Figure 5a–c, Table S3). Thereby, treatment of organoids with S.b.S was found to normalize *Defa1* (*p* < 0.01), *Defa21* (*p* < 0.05), and *Defa5* (*p* < 0.001) gene expression (Figure 5a–c and Figure S2). Similarly, LPS exposure induced *Defa21* expression (*p* < 0.001), whereby these effects were absent when organoids were additionally incubated with S.b.S (*p* < 0.05, Figure 5b and Figure S2). Further analysis of AMPs demonstrated that GF_Red_-induced stress was associated with increased expression of lysozyme (*Lyz1*) (*p* < 0.05) and murine β-defensin 1 (*mBD1*) (*p* < 0.001, Figure 5d,f). However, treatment of organoids with S.b.S reduced *mBD1* expression (*p* < 0.0001, Figure 5f). Notably, LPS-induced stress was also associated with an increase in regenerating islet-derived protein 3 gamma (*Reg3γ*) and *mBD1* gene expression (*p* < 0.05, Figure 5e,f), whereby S.b.S exposition reduced *Reg3γ* (*p* < 0.05) and *mBD1* (*p* < 0.0001) mRNA expression (Figure 5e,f and Figure S2).

**Figure 6:**
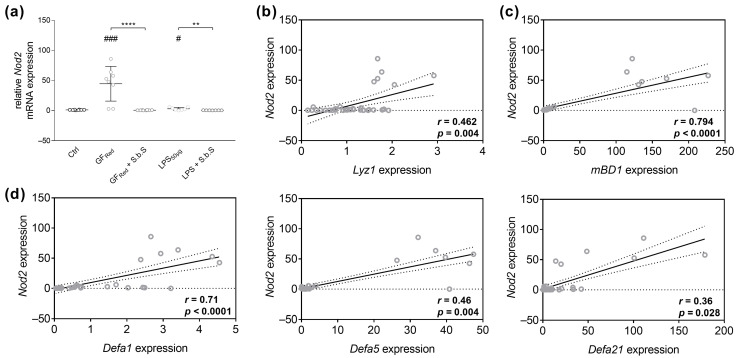
GF_Red_ and LPS-related disturbances in AMP gene expression are correlated with increased Nod2 gene expression in small intestinal organoid cells. Murine small intestinal organoids were treated with S.b.S (200 µg/mL), or GF_Red_ ± S.b.S (200 µg/mL), or LPS (50 µg/mL) ± S.b.S (200 µg/mL), or RPMI as control for 30 h at 37 °C. Gene expression of *Nod2*, *Lyz1*, *mBD1*, *Defa1*, *Defa5*, and *Defa21* was quantified by comparison to standard curves from serially diluted plasmid standards and normalized to β-actin copy numbers. Relative mRNA expression levels of *Nod2* (**a**) determined by quantitative RT-PCR are shown. Data are presented as means ± SEM (n = 8). Statistical analysis was performed by Kruskal–Wallis test with Dunn’s multiple comparisons test (**a**). Differences between two groups were analyzed by using Mann–Whitney test (**a**). Significant differences to RPMI control are indicated as ^#^ *p*-value < 0.05; ^###^ *p*-value < 0.001. Significant differences between two groups are indicated as ** *p*-value < 0.01; **** *p*-value < 0.0001. Correlation analysis for *Nod2* gene expression and *Lyz1* (**b**), *mBD1* (**c**), *Defa1*, *Defa5*, and *Defa21* (**d**) expression. Statistical analysis was performed by two-tailed Spearman rank correlation analysis. Correlations were defined as: 0.2 to 0.4, weak positive correlations; 0.4 to 0.6, moderate positive correlations; 0.6 to 0.8, strong positive correlations. The dashed line represents no correlation (r = 0).

The present study revealed that GF_Red_- (*p* < 0.001) and LPS- (*p* < 0.05) induced stress was associated with an increased *Nod2* mRNA expression (Figure 6a, Table S3), whereby additional treatment of GF_Red_-treated organoids with S.b.S reduced *Nod2* expression (*p* < 0.0001). Similarly, LPS-mediated effects on *Nod2* expression were absent when organoids were co-incubated with S.b.S (*p* < 0.01, Figure 6a and Figure S2). Moreover, spearman rank correlation analysis identified a moderate positive correlation between *Nod2* expression and the expression of *Lyz1* (r = 0.462; *p* = 0.004), *Defa5* (r = 0.46; *p* = 0.004), and *Defa21* (r = 0.36; *p* = 0.028, Figure 6b,d). Further, we found a strong positive correlation between *Nod2* and *mBD1* (r = 0.794; *p* < 0.0001) and *Defa1* expression (r = 0.71; *p* < 0.0001, Figure 6c,d). However, no correlation was found between *Nod2* mRNA levels and the expression of *Reg3γ* (Figure S3).

**Figure 7:**
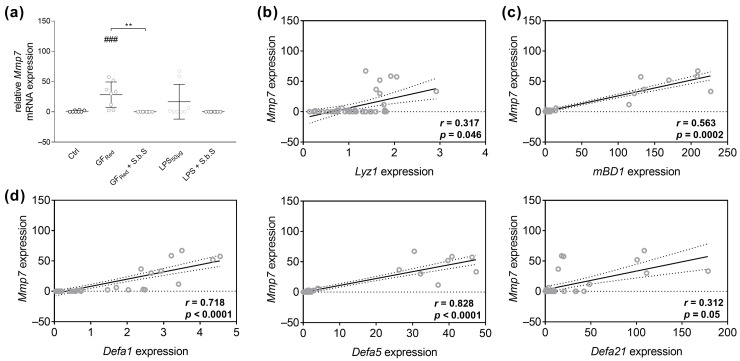
GF_Red_-related disturbances in AMP gene expression are correlated with increased *Mmp7* gene expression. Murine small intestinal organoids were treated with S.b.S (200 µg/mL), or GF_Red_ ± S.b.S (200 µg/mL), or LPS (50 µg/mL) ± S.b.S (200 µg/mL), or RPMI as control for 30 h at 37 °C. Gene expression of *Mmp7*, *Lyz1*, *mBD1*, *Defa1*, *Defa5*, and *Defa21* was quantified by comparison to standard curves from serially diluted plasmid standards and normalized to β-actin copy numbers. Relative mRNA expression levels of *Mmp7* (**a**) determined by quantitative RT-PCR are shown. Data are presented as means ± SEM (n = 8). Statistical analysis was performed using Kruskal–Wallis test with Dunn’s multiple comparisons test (**a**). Differences between two groups were analyzed by using Mann–Whitney test (**a**). Significant differences to RPMI control are indicated as ^###^ *p*-value < 0.001. Significant differences between two groups are indicated as ** *p*-value < 0.01. Correlation analysis for *Mmp7* gene expression and *Lyz1* (**b**), *mBD1* (**c**), *Defa1*, *Defa5*, and *Defa21* (**d**) expression. Statistical analysis was performed by two-tailed Spearman rank correlation analysis. Correlations were defined as 0.2 to 0.4, weak positive correlations; 0.4 to 0.6, moderate positive correlations; 0.6 to 0.8, strong positive correlations; and 0.8 to 1.0, very strong correlations. The dashed line represents no correlation (r = 0).

PCR analysis revealed that stress induction by GF_Red_ resulted in an increase in *Mmp7* gene expression (*p* < 0.001), which was normalized by a concomitant S.b.S treatment (*p* < 0.01, Figure 7a, Table S3). In contrast, LPS-induced stress did not change *Mmp7* mRNA expression (Figure 7a and Figure S2). Moreover, spearman rank correlation analysis revealed a weak positive correlation between *Mmp7* expression and the expression of *Lyz1* (r = 0.317; *p* = 0.046) and *Defa21* (r = 0.312; *p* = 0.05, Figure 7b,d), as well as a moderate positive correlation between *Mmp7* mRNA levels and *mBD1* (r = 0.563; *p* = 0.0002) and a strong positive correlation between *Mmp7* and *Defa1* expression in small intestinal organoids (r = 0.718; *p* < 0.0001, Figure 7c,d). Further, we identified a very strong positive correlation between *Mmp7* and *Defa5* mRNA expression (r = 0.828; *p* < 0.0001) and a weak negative correlation between *Mmp7* and *Reg3γ* (Figure 7d and Figure S3).

### 2.3. S.b.S Reduces Myd88 and Proinflammatory Cytokine Transcript Expression in Stressed Organoids

Analysis of inflammatory responses demonstrated that reduced growth factors (GF_Red_) induced *Myd88* mRNA expression (*p* < 0.001), whereby these effects were no longer present with concomitant S.b.S exposition (*p* < 0.01, Table 1). Increased activation of the TLR/Myd88 signaling pathway has been associated with an induction of proinflammatory cytokine expression, such as *Tnfα* and ILs. Consistently, GF_Red_-induced stress increased *Tnfα* mRNA expression (*p* < 0.001), which was absent when organoids were simultaneously exposed to S.b.S (*p* < 0.001, Table 1). However, LPS incubation did not change *Myd88* or *Tnfα* gene expression (Table 1). Further, GF_Red_-induced stress increased *IL-6* (*p* < 0.01) and *IL-1β* gene expression (*p* < 0.001), whereas LPS exposure had no effects (Table 1). Thereby, additional treatment of GF_Red_-treated organoids with S.b.S decreased *IL-6* (*p* < 0.01) and *IL-1β* (*p* < 0.001) expression (Table 1), implying potential anti-inflammatory effects of S.b.S (Figure S2).

## 3. Discussion

This was the first study investigating the effects of S.b.S in a murine small intestinal organoid model. Using organoid cell culture, we were able to demonstrate that S.b.S (200 µg/mL) improved GF_Red_- and LPS-induced disturbances in TJ and AJ expression and normalized mucus formation, as well as NOD2-mediated AMP formation and MMP7-dependent activation. Further, exposure to supernatant exhibited positive effects on gene expression of several inflammatory markers during stress exposure, supporting the well-known anti-inflammatory characteristics of *S. boulardii*.

We provide evidence that S.b.S treatment regulated stress-induced dysregulation of intestinal barrier markers, particularly by increasing *Muc2*, *Ocln*, and *Cldn7* and by decreasing *Muc1*, *Cldn2*, *Cldn5*, and *JAM-A* expression. These molecular changes are in line with previous studies showing the relevance of intact TJ and mucin formation for intestinal health, and disorders have been associated with GI diseases such as infections (diarrhea) and IBD [37,38]. Specifically, changes in *Cldn2* formation, a pore-forming TJ protein, have been linked with increased water influx into the intestinal lumen in the context of IBD [39]. Further, there is evidence that claudin-5 expression was induced during inflammation via CCAAT/enhancer-binding protein-α in a JAM-A-dependent manner [40]. Similarly, alterations in *Cldn7* gene expression have been associated with GI barrier dysfunction and carcinogenesis [19,41]. Moreover, our results, showing increased *Muc2* and decreased *Muc1* expression upon S.b.S treatment, support previous findings on the distinct roles of mucins in intestinal health. While *Muc1* served as a key element in the host’s pathogen and was found to be induced by proinflammatory cytokines such as Tnfα, IL-6, IL-1β, and IL-22 [42,43], reduction in *Muc2*, on the other hand, has been associated with increased GI barrier permeability [27,44].

During GI infections, pathogens such as *Enteropathogenic Escherichia coli* (*EPEC*) or *Enterohemorrhagic Escherichia coli* (*EHEC*) adhere to the intestinal mucosa and alter TJ structure via effector proteins [45]. In accordance with our results, *EPEC*-infected T84 cells exhibited unchanged transepithelial resistance when exposed to *S. boulardii*. This effect was associated with a preservation of ZO-1 protein levels, suggesting a protective role of *S. boulardii* for TJ structure [46]. Regulation of TJs and anti-inflammatory effects of *S. boulardii* CNCM I-745 were also confirmed for *Shigella* infections [47]. Thus, *S. boulardii* enhanced ZO-2 protein formation and horseradish peroxidase flux across T-84 monolayers, suggesting an improved barrier integrity [47]. Similarly, treatment of cultured colonic explants from IBD patients with *S. boulardii* CNCM I-745 supernatant revealed that S.b.S protected epithelial morphology and maintained cell surface E-cadherin expression. Thereby, recovery of enterocyte AJs was accompanied by an improved Ras-related protein Rab-11A-dependent recycling endosome-dependent restoration [48]. Moreover, 3 months’ supplementation of an oral capsule formulation containing 200 mg lyophilized *S. boulardii*-17 improved intestinal permeability in CD patients by decreasing the lactulose/mannitol ratio [28]. Further, *S. boulardii* ameliorated intestinal barrier integrity in Sprague-Dawley rats with acetic acid-induced colitis by inducing colonic ZO-1 protein expression [49]. Similarly, 3-week administration of *S. boulardii* increased ZO-1 and occludin protein levels in DSS-treated mice, thereby improving intestinal barrier function [50]. This is in line with our data indicating improved mucus formation as well as normalized TJ expression in small intestinal organoids after S.b.S treatment.

Our study showed that GF_Red_ and LPS exposure was associated with increased antimicrobial peptide defense and that *Nod2* and *Mmp7* might be involved. Further S.b.S exposition was found to normalize *Nod2*, *Mmp7*, and AMP gene expression. This is consistent with prior findings showing that murine small intestinal organoids derived from C57BL/6J mice have been shown to contain functional Paneth cells, as evidenced by proteomic and transcriptomic profiling revealing the expression of Paneth cell markers such as lysozyme and CD24. Furthermore, immunostaining confirmed the presence of α-defensins like Crp5 [51,52]. Moreover, it is well known that *S. boulardii* displays antimicrobial properties and potentially even produces antimicrobial proteins. Thus, *S. boulardii* suppressed the virulence of *Citrobacter rodentium*-induced colitis in mice by reducing bacterial adhesion on epithelial cells through the regulation of bacterial effector proteins. This effect might be related to the production of antimicrobial substances [34]. This presumption has been confirmed by in vitro assays testing the efficacy of the cell-free supernatant of *S. boulardii* strains (KT000032, KT000033, KT000034, KT000035, KT000036, and KT000037) on enteropathogenic bacteria. From 13 tested pathogens, *S. boulardii* (KT000032) displayed high antimicrobial activities against several pathogens, like *Enterococcus faecalis*, *Micrococcus luteus*, *Klebsiella pneumoniae*, and *Salmonella typhi* [53]. There is evidence that *S. boulardii* exhibited antimicrobial activity through the release of serine proteases, which are able to cleave microbial toxins A and B of *C. difficile* as well as the enterocytic receptor [13,54]. Furthermore, it has been demonstrated that *S. boulardii* directly produces AMPs. Accordingly, antimicrobial peptides with a low molecular weight (5792 Da) have been isolated from *S. boulardii* ATCC MYA-796TM by ultrafiltration. These purified AMPs showed inhibitory effects against Gram-positive and -negative bacteria, such as *Staphylococcus aureus*, *E. coli*, *Candida albicans*, or *Aspergillus niger* [55].

Our data provides the first evidence that S.b.S modulates host AMP formation. Similar modulating effects on GI antimicrobial peptide defense have already been reported for other probiotics. Hence, treatment of *Pseudomonas aeruginosa*-infected SW480 intestinal epithelial cells with *Lactobacillus rhamnosus* GG or *Bifidobacterium longum* spp. resulted in a NOD1-dependent induction of human β-defensin (*hBD*) 2 expression [56]. This is consistent with our results, suggesting *Nod2* was involved in the regulation of AMP gene expression. Likewise, *E. coli Nissle* 1917 and different *Lactobacilli*, such as *Lactobacillus acidophilus* PZ 1129, *Lactobacillus paracasei*, or *Lactobacillus plantarum*, induced the hBD2 expression by activating hBD2 promoter via nuclear factor k-light-chain-enhancer of activated B cells (NF-κB) in CaCo2 cells [57]. In addition, colonization of germ-free piglets with *E. coli Nissle* 1917 increased small intestinal calprotectin, which might explain the therapeutic effects of *E. coli Nissle* 1917 for inflammatory bowel disease [58]. Microarray analysis in patients with esophagitis also demonstrated that a four-week administration of *Lactobacillus rhamnosus* GG regulated duodenal expression of *Defa1* [59]. Our results point towards the fact that S.b.S might also exert regulatory effects on host antimicrobial peptide defense, with both *Nod2*-dependent induction and *Mmp7*-mediated activation of AMPs being involved. Specifically, S.b.S-mediated downregulation of *Nod2*, an intracellular pattern recognition receptor [60], potentially represents a feedback mechanism limiting immune activation either directly through microbial ligand recognition or indirectly through reduced inflammatory cytokine production. Further, downregulation of *Mmp7* in response to S.b.S also indicates suppression of epithelial immune activation and barrier dysfunction. Consistently, microbiota-released metabolites were found to be regulators of the intestinal immune response. Thus, supplementation with *Flavonifractor plautii*, synthesizing phytosphingosine, increased intestinal phytosphingosine levels in mice after fecal transplantation from phlegm-dampness constitution individuals. Mechanistic studies confirmed that phytosphingosine directly bound to hepatic peroxisome proliferator-activated receptor α, thereby regulating gene expression related to glucose-lipid metabolism. These results support the rationale that microbiota-host signaling and specifically microbiota-secreted factors modulate immune defense and epithelial barrier function [61].

The probiotic yeast *S. boulardii* has been used for decades to treat diarrheal diseases [62,63,64,65,66,67]. It is well known that *S. boulardii* possesses anti-inflammatory activity, including modulation of intracellular signaling pathways associated with inflammatory diseases [68]. In line with this, we demonstrated that S.b.S exerted anti-inflammatory effects, as exposition of GF_Red_- and LPS-treated organoids reduced *Myd88*, *Tnfα*, *IL-6*, and *IL-1β* expression. While GF_Red_- and LPS-treated organoids did not represent a classical IBD model, the observed molecular responses in organoids are mechanistically consistent with previously reported effects of *S. boulardii* in IBD-like conditions. Administration of *S. boulardii* in rats with 2,4,6-trinitrobenzenesulfonic acid (TNBS)-induced colitis improved histological damage, diarrhea, and colonic gene expression of *IL-1β*, *IL-6*, and *Tnfα* [69]. Further, dietary administration of *S. boulardii* to mice with dextran sulfate sodium (DSS)-induced colitis improved colon injury and reduced inflammatory responses by modulating gene expression of proinflammatory cytokines, such as *Tnfα*, *IL-6*, and *IL-1β* [70,71]. These effects were associated with changes in microbiota composition and short-chain fatty acid (SCFA) metabolism, whereby polysaccharides and polypeptides derived from *S. boulardii* promoted the growth of SCFA-producing bacteria [70]. In addition, 12-week treatment with *S. boulardii* improved azoxymethane and DSS-induced ulcerative colitis (UC) carcinogenesis in C57BL/6J mice, which was attributed to a decrease in colon TNFα and IL-6 levels [72]. There is further evidence that microbiota-released metabolites modulate gastrointestinal inflammatory processes and metabolic diseases. Thus, the supplementation of a high-fat and high-sugar diet with the SCFA butyrate in C57BL/6J mice improved intestinal permeability and reduced inflammatory cytokines, such as *IL-6* [20]. Moreover, reduced abundance of *Faecalibacterium prausnitzii* was associated with increased calcification in ApoE^−/−^ mice. Specifically, *Faecalibacterium prausnitzii*-derived butyric acid was identified as a key anti-calcific metabolite, whereby isotope labeling and 13C flux analyses detected butyric acid in the heart tissue, regulating glycolysis-driven calcification by specifically modifying GAPDH [73].

Several studies have demonstrated the immunomodulatory potential of *S. boulardii*. Consistent with our previous results, a rat model of diclofenac-induced enteropathy revealed that *S. boulardii* CNCM I-745 prevented TLR2/4, MYD88, and NF-κB p65 overexpression, thereby decreasing proinflammatory cytokines, such as IL-1β [10]. Cultivation of purified LPS-stimulated dendritic cells (DC) CD1c + CD11c + CD123-myeloid DC (mDC) from patients with UC or CD revealed that S.b.S exposition reduced frequency of CD40-, CD80- and CD197-expressing mDC, as well as decreased DC-secretion of TNFα and IL-6. In addition, S.b.S inhibited IBD mDC-induced T-cell proliferation and UC mDC-induced TH1 polarization via TNFα and interferon-γ (IFN-γ) and promoted IL-8- and transforming growth factor-β-dependent mucosal healing [9]. Similarly, in C.B-17 SCID mice with IBD induced by intraperitoneal 4 × 10^5^ CD4 + CD45RBhi T-cell injection, oral administration of *S. boulardii* reduced colonic inflammation, which was associated with decreased colonic NF-κB activity and lower expression of proinflammatory cytokines. Administration of *S. boulardii* specifically modulated IFN-γ production of CD4+ T-cells, accompanied by downregulation in the colon and upregulation in mesenteric lymph nodes, indicating a potential redistribution of IFN-γ-producing T-cells [74]. Oral administration of *S. boulardii* further improved weight loss and histologic injury and preserved the colon barrier in mice with DSS-induced colitis. Thereby, *S. boulardii* specifically inhibited DSS-dependent upregulation of hypoxia-induced factors -1α and -2α, resulting in reduced expression of epithelial-mesenchymal transition markers E-cadherin and vimentin and of vascular endothelial growth factor [67]. Our study revealed novel mechanistic evidence that S.b.S regulated intestinal barrier markers and immune signaling in small intestinal organoids, strongly supporting the well-known anti-inflammatory effects of the *S. boulardii* CNCM I-745 strain.

However, future studies are needed to confirm these effects in vivo and under disease-specific conditions. While these findings strengthen the rationale for using *S. boulardii* CNCM I-745 as a supportive therapy in diarrheal diseases, several limitations of the present study have to be confessed. First, the organoid model, although physiologically relevant, lacks lamina propria immune cells and microbial components of the GI tract. The complexity of the GI tract requires further investigation to completely elucidate the molecular mechanisms of S.b.S. Second, the current study focused on functional outcomes in a 3D organoid model; additional mechanistic studies, such as signaling pathway analyses and proteomic profiling, could improve the understanding of the molecular mechanisms involved in S.b.S-mediated effects. Further, we did not analyze AMP protein levels or MMP7 enzymatic activity. While our data indicate increased transcription of *Mmp7* and *Defa5*, it remains to be verified whether these changes are associated with enhanced antimicrobial activity. Although increased *Mmp7* has been shown to activate Paneth cell pro-α-defensins into antimicrobial active mature peptides [29], further studies including *Mmp7* activity assays as well as mechanistic experiments using specific inhibitors, gene knockdown strategies, or CRISPR/Cas9-based approaches targeting *Nod2* and *Mmp7* are needed. Protein-level confirmation by ELISA or Western blotting for key cytokines and AMPs will clarify the role of S.b.S in host defense modulation. Moreover, future research should aim to identify the molecular components responsible for the observed effects, for instance, through fractionation and proteomic analyses. Additionally, preclinical in vivo studies are required to validate the efficacy and safety of S.b.S in disease-relevant models of intestinal inflammation or infection. Furthermore, understanding the molecular mechanisms and active ingredients of S.b.S could support the development of novel microbiome-based or postbiotic therapies. Further studies are necessary to assess efficacy and safety in murine models of intestinal inflammation and infection, alongside fractionation approaches to identify bioactive components responsible for the observed effects.

## 4. Materials and Methods

### 4.1. Generation of Saccharomyces boulardii Supernatant

S.b.S was prepared under standardized conditions from S. boulardii CNCM I-745. A 10% weight/volume *S. boulardii* CNCM I-745 (100 mg/mL, Perenterol^®^ forte, BIOCODEX, Batch No.: 1247, produced in February 2023, Gentilly, France) suspension was prepared by resuspending in 25 mL Roswell Park Memorial Institute (RPMI) 1640 medium (Merck, Darmstadt, Germany) in a 100 mL Erlenmeyer flask. After aerobic incubation at 37 °C and 200 rpm for 24 h (HT Infors Minitron AI 71, Infors AG, Bottmingen, Switzerland), the suspension was centrifuged at 10,000× *g* for 10 min (Centrifuge 5417R, M&S, Wiesbaden, Germany). The centrifugation step was repeated until the supernatant was clear. Finally, the S. boulardii pellet was removed, and the obtained supernatant (referred to as S.b.S) was filtered sterile (0.22 µm, polyvinylidene fluoride filter, Carl Roth GmbH, Karlsruhe, Germany).

### 4.2. BCA Protein Assay

For determination of S.b.S protein levels, a BCA protein assay was performed (Thermo Scientific, Rockford, IL, USA). Therefore, 8 BSA standards with a detection range of 25 µg/mL to 2000 µg/mL were prepared. A total of 25 µL of standard and 25 µL of S.b.S samples (n = 4), as well as 200 µL of working reagent, were added to a 96-well plate and then incubated at 37 °C for 30 min. After cooling to room temperature, standards, samples, and water as a control were measured at 562 nm on a microplate absorbance reader (BioTek Instruments, Winooski, VT, USA).

### 4.3. Organoid Cell Culture

#### 4.3.1. Isolation and Cultivation

For generating intestinal organoids, crypts from the small intestine of C57BL/6J mice were isolated by incubation at 4 °C for 25 min with crypt isolation buffer (CIB, PBSO containing 0.5 M EDTA). Isolated crypts were counted, and a total of 500 crypts were plated in 25 µL Matrigel (Corning B.v., Amsterdam, The Netherlands), and 300 µL of crypt culture medium (CCM) consisting of advanced DMEM/F12 (ThermoFisher Scientific, Karlsruhe, Germany) supplemented with 100 ng/µL Noggin (PeproTech, East Windsor, NJ, USA), 1 µg/mL R-Spondin (PeproTech, East Windsor, NJ, USA), B-27™ supplement 1× (Invitrogen, Carlsbad, CA, USA), 1 mM N-Acetylcysteine (Sigma-Aldrich, Schnelldorf, Germany), 0.1 mg/mL Primocin (Invitrogen, Carlsbad, CA, USA) and 50 ng/mL mEGF (Immunotools, Friesoythe, Germany) were added. The resulting organoids were cultured for a minimum of seven days according to Sato et al. 2009 [75]. Organoid growth was monitored by light microscopy.

#### 4.3.2. Media Change and Cell Passage

Cell culture medium was changed every 3rd day, and organoids were passaged 1:5. Therefore CCM was replaced by 500 µL wash buffer (advanced DMEM/F12), containing Pen (100 U/mL)/Strep (100 µg/mL) (ThermoFisher Scientific, Karlsruhe, Germany) and 7.5% BSA (solved in PBSO; Carl Roth GmbH, Karlsruhe, Germany). Organoids were mechanically disrupted and centrifuged at 200 g for 5 min (Megafuge 1.0, M&S, Wiesbaden, Germany). Dissociated organoids were washed with 2 mL wash buffer and centrifuged at 200 g for 5 min (Megafuge 1.0, M&S, Wiesbaden, Germany). Organoid pellets were suspended with 25 µL Matrigel, plated in a 48-well plate, and 300 µL CCM were added.

### 4.4. Dose-Finding Studies

For dose determination, the effects of different S.b.S concentrations on cell number and viability were investigated by using an MTT assay (n = 4). Further, markers of GI barrier and inflammation were analyzed by RT-PCR (n = 4).

#### 4.4.1. MTT-Assay

Organoids were incubated with 4.6 µL S.b.S (dissolved in RPMI, stock solution 3 mg/mL for a final concentration of 200 µg/mL in 70 µL CCM, n = 4), or 1.5 µL S.b.S (dissolved in RPMI, stock solution 3 mg/mL for a final concentration of 67 µg/mL in 70 µL CCM, n = 4), or 0.46 µL S.b.S (dissolved in RPMI, stock solution 3 mg/mL for a final concentration of 20 µg/mL in 70 µL CCM, n = 4), or with a corresponding amount of RPMI as a control (n = 4) at 37 °C for 30 h. A total of 7 µL of MTT solution (500 mg/mL, dissolved in PBSO) was added, and after incubation for 1 h at 37 °C, 5% CO_2_, CCM was discarded, and cells were incubated with 20 µL of SDS (for 1 h at 37 °C, 5% CO_2_) to solubilize the Matrigel. Finally, 100 µL DMSO was added (for 1 h at 37 °C, 5% CO_2_), and optical density was measured at 562 nm on a microplate absorbance reader (BioTek Instruments, Winooski, VT, USA).

#### 4.4.2. RT-PCR

To examine the effects of S.b.S on the GI barrier and inflammatory markers, gene expression was analyzed by RT-PCR (FXConnectTM Real-Time Systems, BioRad Laboratories, Munich, Germany). For this purpose, organoids were exposed to 20 µL S.b.S (dissolved in RPMI, stock solution 3 mg/mL for a final concentration of 200 µg/mL in 300 µL CCM, n = 4), or to 6.7 µL S.b.S (dissolved in RPMI, stock solution 3 mg/mL for a final concentration of 67 µg/mL in 300 µL CCM, n = 4), or to 2 µL S.b.S (dissolved in RPMI, stock solution 3 mg/mL for a final concentration of 20 µg/mL in 300 µL CCM, n = 4), or to a corresponding amount of RPMI as a control (n = 4) at 37 °C for 30 h. From organoids, total RNA was extracted using the ExtractME Total RNA Kit (blirt S.A, Hilden, Germany). RNA quality was assessed by measuring the A260/A280 ratio using a NanoDrop spectrophotometer (Peqlab Biotechnologie, Erlangen, Germany). Samples were considered of high purity with ratios between 1.8 and 2.0, and RNA was reverse-transcribed in a thermocycler (BioRad Laboratories, Munich, Germany) using the Reverse Transcription System kit and random primers after a DNase digestion step (Promega, Madison, WI, USA). For RT-PCR analysis, primer working solutions of 1 ng/μL were prepared by diluting 20 μL primer stock (100 pmol/μL) with 180 μL ultrapure water. Relative gene expression of *Tnfα*, *Myd88*, *Cldn7*, and *Ocln* was calculated by comparison to the housekeeping gene β-actin using the ΔΔ−Ct method. The oligonucleotide primer sequences are listed in Table S4.

### 4.5. Stimulation

To examine the effects of S.b.S during cell stress on GI barrier and inflammation, organoids were incubated with modified CCM (reduced growth factors, GF_Red_, n = 8, Table 2) or with LPS (50 µg/mL, n = 8). Organoids were treated with 15.99 µL S.b.S (dissolved in RPMI, stock solution 3.753 mg/mL for a final concentration of 200 µg/mL in 300 µL CCM, n = 8), or GF_Red_ ± S.b.S (200 µg/mL, n = 8), or LPS (50 µg/mL) ± S.b.S (200 µg/mL, n = 8), or the corresponding amount of RPMI as a control (n = 8) at 37 °C for 30 h.

### 4.6. RNA Isolation, Generation of Standard Plasmids, and RT-PCR

Total RNA was isolated from organoids using the EXTRACTME Total RNA Kit (blirt S.A). RNA quality was assessed by measuring the A260/A280 ratio using a NanoDrop spectrophotometer (Peqlab Biotechnologie, Erlangen, Germany). Samples were considered of high purity with ratios between 1.8 and 2.0 and synthesized to complementary cDNA using the Reverse Transcription System (Promega, Madison, WI, USA). To analyze absolute gene expression, standard plasmids were generated by using the TOPO TA Cloning^®^Kit For Sequencing (lifetechnologies™, Carlsbad, CA, USA). Target genes were amplified, placed into a plasmid vector, and transformed into competent One Shot^®^TOP10 + DH5α™-T1^®^ cells (Invitrogen, Carlsbad, CA, USA). Characterization of plasmid DNA was carried out by sequencing (GATC Biotech AG, Konstanz, Germany). For RT-PCR analysis, primer working solutions of 1 ng/μL were prepared by diluting 20 μL primer stock (100 pmol/μL) with 180 μL ultrapure water. Absolute gene expression of *Defa1*, *Defa21*, *Defa5*, *mbD1*, *Lyz1*, *Reg3γ*, *Nod2*, and *Mmp7* was determined by comparison with a quantitative standard curve generated by serial dilution of plasmid standards and normalized to the copy numbers of the mouse housekeeping gene β-actin. Relative gene expression of *ZO-1*, *JAM-A*, *Cldn2*, *Cldn5*, *Cldn7*, *Ocln*, *Muc1*, *Muc2*, *IL-6*, *IL-1β*, *Myd88*, and *Tnfα* was calculated by comparison to the housekeeping gene β-actin using the ΔΔ−Ct method. The oligonucleotide primer sequences are listed in Table S4.

### 4.7. Statistical Analysis

All statistical analyses were performed using GraphPad Prism software 7.0 (GraphPad Software Inc., La Jolla, CA, USA). Normal distribution was analyzed using the Kolmogorov-Smirnov test, and outliers were identified by the ROUT method (Q = 1%). For statistical comparison of more than two groups, a one-way ANOVA with Dunnett’s multiple comparisons test or Kruskal–Wallis test with Dunn’s multiple comparisons test was performed. Differences between two groups were analyzed by using the unpaired *t*-test or the Mann–Whitney test. *p*-values at <0.05 were considered statistically significant. A statistical trend was defined as 0.05 > *p*-value < 0.1. Data are shown as mean ± SEM. Correlation analyses were performed with two-tailed Spearman rank correlation, with coefficients in the range of 0.0 to 0.2 (0.0 to −0.2) defined as no correlation, in the range of 0.2 to 0.4 or −0.2 to −0.4 defined as weak positive or negative correlation, in the range of 0.4 to 0.6 or −0.4 to −0.6 defined as moderate positive or negative correlation, in the range of 0.6 to 0.8 or −0.6 to −0.8 defined as strong positive or negative correlation, and in the range of 0.8 to 1.0 or −0.8 to −1.0 defined as very strong positive or negative correlation.

## 5. Conclusions

In conclusion, our data demonstrated that S.b.S at 200 µg/mL, 67 µg/mL, and 20 µg/mL was appropriate for use in murine small intestinal organoids. Exposure of organoids to S.b.S at a concentration of 200 µg/mL exhibited positive effects on inflammatory markers and stress-induced disturbances in TJ, AJ, and mucin expression. Moreover, S.b.S exposition normalized antimicrobial peptide defense, whereby *Nod2* and *Mmp7* might be involved. Thus, the present study provides new insights on molecular mechanisms by which products released by *S. boulardii* CNCM I-745 exert anti-inflammatory properties and the capability to modulate GI barrier function during stress conditions.

## Figures and Tables

**Table 1:** Inflammatory transcripts measured in small intestinal organoids.

	*Myd88*	*Tnfα*	*IL-6*	*IL-1β*
Ctrl	2.44 ± 0.71	1.49 ± 0.28	1.21 ± 0.22	4.41 ± 1.72
GF_Red_	58.05 ± 12.69 ^###^	46.58 ± 12.02 ^###^	134.4 ± 36.97 ^##^	66.39 ± 16.45 ^###^
GF_Red_ ± S.b.S	11.76 ± 2.98 **	0.3 ± 0.1 ***	0.79 ± 0.37 **	5.88 ± 1.22 ***
LPS	23.05 ± 14.18	20.87 ± 12.37	42.24 ± 25.97	42.8 ± 28.17
LPS ± S.b.S	14.5 ± 1.8 ^$^	0.6 ± 0.17	0.79 ± 0.13 ^$^	4.38 ± 0.61

S.b.S at 200 µg/mL reduces GF_Red_-mediated induction of the *Myd88* and proinflammatory cytokines. Murine small intestinal organoids were treated with S.b.S (200 µg/mL), or GF_Red_ ± S.b.S (200 µg/mL), or LPS (50 µg/mL) ± S.b.S (200 µg/mL), or RPMI as control for 30 h at 37 °C. Gene expression of *Myd88*, *Tnfα*, *IL-6*, and *IL-1β* was assessed by RT-PCR using the ΔΔCt method normalized to β-actin. Relative mRNA expression levels of *Myd88*, *Tnfα*, *IL-6*, and *IL-1β* determined by quantitative RT-PCR are shown. Data are presented as means ± SEM (n = 8). Statistical analysis was performed using Kruskal–Wallis test with Dunn’s multiple comparisons test. Differences between two groups were analyzed by using Mann–Whitney test. Significant differences to RPMI control are indicated as ^##^ *p*-value < 0.01; ^###^ *p*-value < 0.001. Significant differences to GF_Red_ are indicated as ** *p*-value < 0.01; *** *p*-value < 0.001. Significant differences to LPS are indicated as ^$^ *p*-value < 0.05.

**Table 2:** Modifications of cell culture medium (GF_Red_).

	CCM	GF_Red_
GlutaMax^TM^	2 mM	2 mM
Hepes	10 mM	10 mM
R-Spondin	1 µg/mL	0.5 µg/mL
Noggin	100 ng/µL	50 ng/µL
B-27™ supplement	20 µL/mL	20 µL/mL
N-Acetylcysteine	1.63 mg/mL	1.63 mg/mL
Primocin	0.1 mg/mL	0.1 mg/mL
mEGF	50 ng/mL	50 ng/mL

## Data Availability

The data presented in this study are available upon justified request to the corresponding author.

## Supplementary Materials

The following supporting information can be downloaded at: https://www.mdpi.com/article/10.3390/ph18081167/s1, Figure S1: Determination of protein concentration in S.b.S by BCA protein assay; Figure S2. Heatmap of relative gene expression levels of tight junction proteins, mucins, antimicrobial peptides, and inflammatory markers in murine small intestinal organoids; Figure S3: Correlation analysis for *Nod2* (a) or *Mmp7* (b) and *Reg3γ* mRNA expression; Table S1: Cell survival in % calculated by MTT assay; Table S2: Cell number calculated by MTT as-say; Table S3. RT-PCR genes with fold-change and significance; Table S4: Primers used for RT-PCR; COA 01: 64960_1247_COC_EN_V01; COA 02: 64960_1247_COC_EN_V02.

## Abbreviations

The following abbreviations are used in this manuscript:

S.b.S	*Saccharomyces boulardii* supernatant
GI	Gastrointestinal
LPS	Lipopolysaccharide
GF_Red_	Cell culture medium with reduced growth factors
MTT	3-[4,5-dimethylthiazol-2-yl]-2,5 diphenyl tetrazolium bromide
TJ	Tight junction
AJ	Adherent junction
Muc	Mucin
Ocln	Occludin
Cldn	Claudin
ZO-1	Zonula occludens 1
JAM-A	Junctional adhesion molecule A
Nod	Nucleotide binding oligomerization domain
Mmp7	Matrix metalloproteinase-7
Myd88	Myeloid differentiation primary response 88
IL	Interleukin
Tnfα	Tumor necrosis factor α
*S. boulardii*	*Saccharomyces boulardii*
*C. difficile*	*Clostridioides difficile*
*E. coli*	*Escherichia coli*
IBD	Inflammatory bowel disease
AMP	Antimicrobial peptide
CD	Crohn’s disease
Ctrl	RPMI control
ANOVA	One-way analysis of variance
SEM	Standard error of the mean
Defa	α-defensin
Lyz1	Lysozyme
mBD1	Murine β-defensin 1
Reg3γ	Regenerating islet-derived protein 3 gamma
PRR	Pattern recognition receptor
TLR	Toll-like receptor
*EPEC*	*Enteropathogenic Escherichia coli*
*EHEC*	*Enterohemorrhagic Escherichia coli*
hBD	Human β-defensin
NF-κB	Nuclear factor k-light-chain-enhancer of activated B cells
TNBS	2,4,6-Trinitrobenzenesulfonic acid
DSS	Dextran sulfate sodium
SCFA	Short chain fatty acid
UC	Ulcerative colitis
DC	Dendritic cell
mDC	Myeloid DC
IFN-γ	Interferon-γ
RPMI	Roswell Park Memorial Institute
CIB	Crypt isolation buffer
CCM	Cell culture medium
