# Supplementary material for: Saccharomyces boulardii CNCM I-745 Supernatant Improves Markers of Gut Barrier Function and Inflammatory Response in Small Intestinal Organoids
Source: Pharmaceuticals (Basel). 2025 Aug 6;18(8):1167. doi: 10.3390/ph18081167 (PMC12389396; doi:10.3390/ph18081167)
Supplement: Supplementary file 1 [file pharmaceuticals-18-01167-s001.zip › 64960_1247_COA_EN_V02.PDF]

BIOCODEX
healthcare with passion and conviction

CERTIFICATE OF ANALYSIS

Quality Department - 1, Avenue Blaise Pascal
60000 Beauvais - France

PRODUCT IDENTIFICATION

Specification number (MGE) / Version	**SR000592 / 1**	Analysis folder number	**X00018378**
Product name	**PERENTEROL FORTE 250 mg 20 capsules**<br>*PERENTEROL F 250 MG 20 GÉL ALLEMAGNE*		
Product Code (SKU number)	**64960**	Batch number	**1247**

RESULTS

Test	Method	Specification	Result	Unit
Appearance	MCQ000028	Cream white powder in opaque white capsule, with characteristic yeast odor.	Complies	-
Microscopy	MCQ000229	Ovoid yeasts cells. Terminal or subterminal buds are frequently observed.	Complies	-
Study of the assimilation of sugars and other carbohydrate products (API 20C AUX)	MCQ000346	2000032<br>2000072<br>6000032<br>6000072	Complies	-
Mean mass	MCQ000016	257 to 314	286	mg
Uniformity of mass	MCQ000016	Complies Eur. Ph. 2.9.5.	Complies	-
Disintegration time	MCQ000031	<30	5	min
Water content (K.F)	MCQ000331	≤ 2	2	%
Viability determination	MCQ000229	≥ 5	11	10^9cells/u
Assay of total nitrogen on the intermediate product	MCQ000335	5.0 to 7.5	6.0	% N
Active substance assay	MCQ000335	254 to 311	279	mg/unit
AMCC method I	MCQ000347	≤ 1000	< 10	CFU/g
YMCC method II	MCQ000350	≤ 100	< 100	CFU/g
Enterobacteriaceae and bile tolerant gram-negative bacteria	MCQ000090	Absence/g	Complies	-
Escherichia coli	MCQ000341	Absence/g	Complies	-
Salmonella	MCQ000342	Absence/10 g	Complies	-
Pseudomonas aeruginosa	MCQ000343	Absence/g	Complies	-
Staphylococcus aureus	MCQ000344	Absence/g	Complies	-

CONCLUSION

It is hereby certified that the above information is authentic and accurate and that the analysis records have been reviewed and found in compliance with the above specification.

QC qualified person **SAINT GENIS Jérémy**

Signature

Date 12-Apr-2023 17:36:32
