# Supplementary material for: Saccharomyces boulardii CNCM I-745 Supernatant Improves Markers of Gut Barrier Function and Inflammatory Response in Small Intestinal Organoids
Source: Pharmaceuticals (Basel). 2025 Aug 6;18(8):1167. doi: 10.3390/ph18081167 (PMC12389396; doi:10.3390/ph18081167)
Supplement: Supplementary file 1 [file pharmaceuticals-18-01167-s001.zip › 64960_1247_COC_EN_V01.pdf]

BIOCODEX

Usine : 1 Avenue Blaise Pascal
60000 Beauvais, France
Tel. : 33 (0)3 44 02 03 12
Fax : 33 (0)3 44 84 77 49

CERTIFICATE OF COMPLIANCE

Product name : PERENTEROL F 250 MG 20 GEL ALLEMAGNE

Product code : 64960

Batch number : 1247

Released quantity : 62 730 UNITS

Manufacturing Date : 02 2023

Expiry date : 01 2026

I hereby certify that the above information is authentic and accurate. This batch of product has been manufactured and tested in compliance with the EU cGMP requirements and with the specifications of the Marketing Authorisation.

Date :

BIOCODEX
Dr Kevin MARCHE
Pharmacien Libérateur - Qualifed Person
19 AVR. 2023

Qualified Person Signature :
