# Supplementary material for: Saccharomyces boulardii CNCM I-745 Supernatant Improves Markers of Gut Barrier Function and Inflammatory Response in Small Intestinal Organoids
Source: Pharmaceuticals (Basel). 2025 Aug 6;18(8):1167. doi: 10.3390/ph18081167 (PMC12389396; doi:10.3390/ph18081167)
Supplement: Supplementary file 1 [file pharmaceuticals-18-01167-s001.zip › pharmaceuticals-3747980 Supplements_reviesed.docx]

Supplements

Table S1. Cell survival in % calculated by MTT assay.

|  | **RPMI control** | **S.b.S (200 µg/ml)** | **S.b.S (67 µg/ml)** | **S.b.S (20 µg/ml)** |
| --- | --- | --- | --- | --- |
| Replicate 1 | 114.3658 | 78.20603 | 102.3125 | 117.7295 |
| Replicate 2 | 85.21374 | 101.7519 | 97.82726 | 80.44849 |
| Replicate 3 | 85.49404 | 66.15277 | 79.32726 | 102.0322 |
| Replicate 4 | 114.9264 | 150.5256 | 98.38823 | 93.06237 |
| Mean | 100 | 99.16 | 94.46 | 98.32 |
| Std. deviation | 16.91 | 37.3 | 10.29 | 15.68 |
| Std. error of mean | 8.457 | 18.65 | 5.143 | 7.84 |

Table S2. Cell number calculated by MTT assay.

|  | **RPMI control** | **S.b.S (200 µg/ml)** | **S.b.S (67 µg/ml)** | **S.b.S (20 µg/ml)** |
| --- | --- | --- | --- | --- |
| Replicate 1 | 1801.261 | 1231.745 | 1611.423 | 1854.24 |
| Replicate 2 | 1342.116 | 1602.593 | 1540.785 | 1267.064 |
| Replicate 3 | 1346.531 | 1041.906 | 1249.404 | 1607.008 |
| Replicate 4 | 1810.091 | 2370.778 | 1549.615 | 1465.732 |
| Mean | 1575 | 1562 | 1488 | 1549 |
| Std. deviation | 266.4 | 587.5 | 162 | 247 |
| Std. error of mean | 133.2 | 293.7 | 81.01 | 123.5 |

Table S3. RT-PCR genes with fold-change and significance

|  | RPMI control | GF_Red_ | GF_Red_ ± S.b.S | LPS_50µg_ | LPS_50µg_ ± S.b.S |
| --- | --- | --- | --- | --- | --- |
| *ZO-1* | 1.352 ± 0.3882 | 0.7056 ± 0.08031 | 0.8137 ± 0.0984 | 0.589 ± 0.1777 | 0.5566 ± 0.0583 |
| *Ocln* | 1843 ± 0.6033 | **0.3748 ± 0.09146 #** | **3.479 ± 0.3054 ****** | **0.2282 ± 0.1208 #** | **2821 ± 0.1509 $$$$** |
| *JAM-A* | 1.046 ± 0.1064 | **7.702 ± 1.883 ##** | **1.185 ± 0.1148 **** | 3.27 ± 1.647 | 1.244 ± 0.0714 |
| *Cldn2* | 1.443 ± 0.4589 | 2.818 ± 0.6202 | **0.7402 ± 0.06678 **** | 1.25 ± 0.5163 | **0.283 ± 0.04996 $** |
| *Cldn5* | 1.295 ± 0.2849 | **74.07 ± 23.02 ###** | **1.068 ± 0.1958 **** | 46.47 ± 31.01 | **0.6594 ± 0.06434 $** |
| *Cldn7* | 1.009 ± 0.2644 | **0.1331 ± 0.06574 #** | **1.081 ± 0.3108 *** | **0.2993 ± 0.1677 #** | **0.9158 ± 0.1665 $$** |
| *Muc1* | 1.177 ± 0.2788 | **136.4 ± 35.05 ##** | **0.2631 ± 0.0148 **** | 4.816 ± 3.335 | 0.2272 ± 0.05439 |
| *Muc2* | 1.199 ± 0.2999 | **0.2176 ± 0.09315 ##** | **0.9687 ± 0.1347 *** | 0.9366 ± 0.267 | 1.525 ± 0.218 |
| *Defa1* | 0.9988 ± 0.391 | **2.81 ± 0.4646 #** | **0.1625 ± 0.01411 **** | **1.424 ± 0.4439 ###** | **0.07625 ± 0.0127 $** |
| *Defa21* | 1 ± 0.158 | **60.54 ± 22.42 #** | **7.106 ± 0.8319 *** | **32.69 ± 11.97 ###** | **4.41 ± 0.7875 $** |
| *Defa5* | 1.003 ± 0.1277 | **29.29 ± 6.284 ####** | **1.437 ± 0.1399 ***** | 9.676 ± 5.765 | 0.7275 ± 0.09978 |
| *Lyz1* | 0.9988 ± 0.1529 | **1.699 ± 0.2253 #** | 1.241 ± 0.18 | 1.325 ± 0.1915 | **0.6388 ± 0.0879 $$** |
| *Reg3γ* | 1 ± 0.2764 | 1.019 ± 0.4357 | 0.76 ± 0.1327 | **10.72 ± 3.582 #** | **1.706 ± 0.2533 $** |
| *mBD1* | 1.001 ± 0.2133 | **114.2 ± 26.54 ###** | **0.4425 ± 0.0411 ****** | **57.89 ± 32.93 #** | **0.311 ± 0.044 $$$$** |
| *Nod2* | 0.9988 ± 0.1542 | **44.54 ± 10.18 ###** | **0.505 ± 0.09582 ****** | **2.897 ± 0.9104 #** | **0.3586 ± 0.0291 $$** |
| *Mmp7* | 1 ± 0.4851 | **28.31 ± 7.468 ###** | **0.02875 ± 0.0044 **** | 16.7 ± 10.14 | 0.01875 ± 0.003504 |

Murine small intestinal organoids were treated with S.b.S (200 µg/mL), or GF_Red_ ± S.b.S (200 µg/mL), or LPS (50 µg/mL) ± S.b.S (200 µg/mL) or RPMI as control for 30 h at 37°C. Gene expression of *ZO-1, Ocln, JAM-A, Cldn2, Cldn5, Cldn7, Muc1, Muc2, Defa1, Defa21, Defa5, Lyz1, Reg3γ, mBD1, Nod2*, and *Mmp7* was assessed by RT-PCR. Data are presented as means ± SEM (n = 8). Statistical analysis was performed by one-way ANOVA with Dunnett's multiple comparisons test or Kruskal-Wallis test with Dunn's multiple comparisons test. Differences between two groups were analyzed by using unpaired t-test or Mann–Whitney test. Significant differences to RPMI control are indicated as #p-value < 0.05; ##p-value < 0.01; ###p-value < 0.001, ####p-value < 0.0001. Significant differences to GF_Red_ are indicated as *p-value < 0.05; **p-value < 0.01; ***p-value < 0.001; ****p-value < 0.0001. Significant differences to LPS are indicated as $p-value < 0.05; $$p-value < 0.01; $$$$p-value < 0.0001.

Table S4. Primers used for RT-PCR.

| **Primer** | **Forward (5’-3’)** | **Reverse (5’-3’)** |
| --- | --- | --- |
| *Actb* | GCTGAGAGGGAAATCGTGCGTG | CCAGGGAGGAAGAGGATGCGG |
| *Defa1* | TCAAGAGGCTGCAAAGGAAGAGAAC | TGGTCTCCATGTTCAGCGACAGC |
| *Defa5* | TCAAAAAAGCTGATATGCTATTG | AGCTGCAGCAGAATACGAAAG |
| *Defa21* | CCAGGGGAAGATGACCAGGCT | TGCAGCGACGATTTCTACAAAGGC |
| *Lyz1* | GCCAAGGTCTACAATCGTTGTGAGTTG | CAGTCAGCCAGCTTGACACCACG |
| *Reg3γ* | TTCCTGTCCTCCATGATCAAAA | CATCCACCTCTGTTGGGTTCA |
| *mBD1* | CCAGGCTGCCCATCTAATACC | ATTTCCTGCTCGCTACCTAA |
| *Mmp7* | TTCAAGAGGGTTAGTTGGGGGACTG | CCGCCTCTACGAGTGAAACTGTT |
| *Nod2* | GGCACCTGAAGTTGACATTTTGC | ATCTCCCACAGAGTTGTAATCC |
| *Muc1* | TCCTTCTGAGAGCCACCACT | GGTGACCACTTCTGCCAACT |
| *Muc2* | GATGGCACCTACCTCGTTGT | GTCCTGGCACTTGTTGGAAT |
| *Cldn2* | GTCATCGCCCATCAGAAGAT | ACTGTTGGACAGGGAACCAG |
| *Cldn5* | GCTCTCAGAGTCCGTTGACC | CTGCCCTTTCAGGTTAGCAG |
| *Cldn7* | GCGACAACATCATCACAGCC | CCTTGGAGGAATTGGACTTGG |
| *JAM-A* | CACCTTCTCATCCAGTGGCATC | CTCCACAGCATCCATGTGTGC |
| *Ocln* | ACTCCTCCAATGGACAAGTG | CCCCACCTGTCGTGTAGTCT |
| *ZO-1* | CCACCTCTGTCCAGCTCTTC | CACCGGAGTGATGGTTTTCT |
| *IL-6* | AGTCACAGAAGGAGTGGCTA | CTGACCACAGTGAGGAATGT |
| *IL-1β* | ACGGATTCCATGGTGAAGTC | GAGTGTGGATCCCAAGCAAT |
| *Myd88* | CAAAAGTGGGGTGCCTTTGC | AAATCCACAGTGCCCCCAGA |
| *Tnfα* | ACCACCATCAAGGACTCA | AGGTCTGAAGGTAGGAAG |

Abbreviation: *Actb*, β-Actin; *Defa*, α-defensin; *Lyz1*, Lysozyme; *Reg3γ*, Regenerating islet-derived protein 3 gamma; *mBD1*, Murine β-defensin 1; *Mmp7*, Matrix metalloproteinase-7; *Nod2*, Nucleotide binding oligomerization domain; *Muc*, Mucin; *Cldn*, Claudin; *JAM-A*, Junctional adhesion molecule A; *Ocln*, Occludin, *ZO-1*, zonula occludens 1; *IL*, Interleukin; *Myd88*, Myeloid differentiation primary response 88; *Tnfα*, Tumor necrosis factor α.

Figure S1:


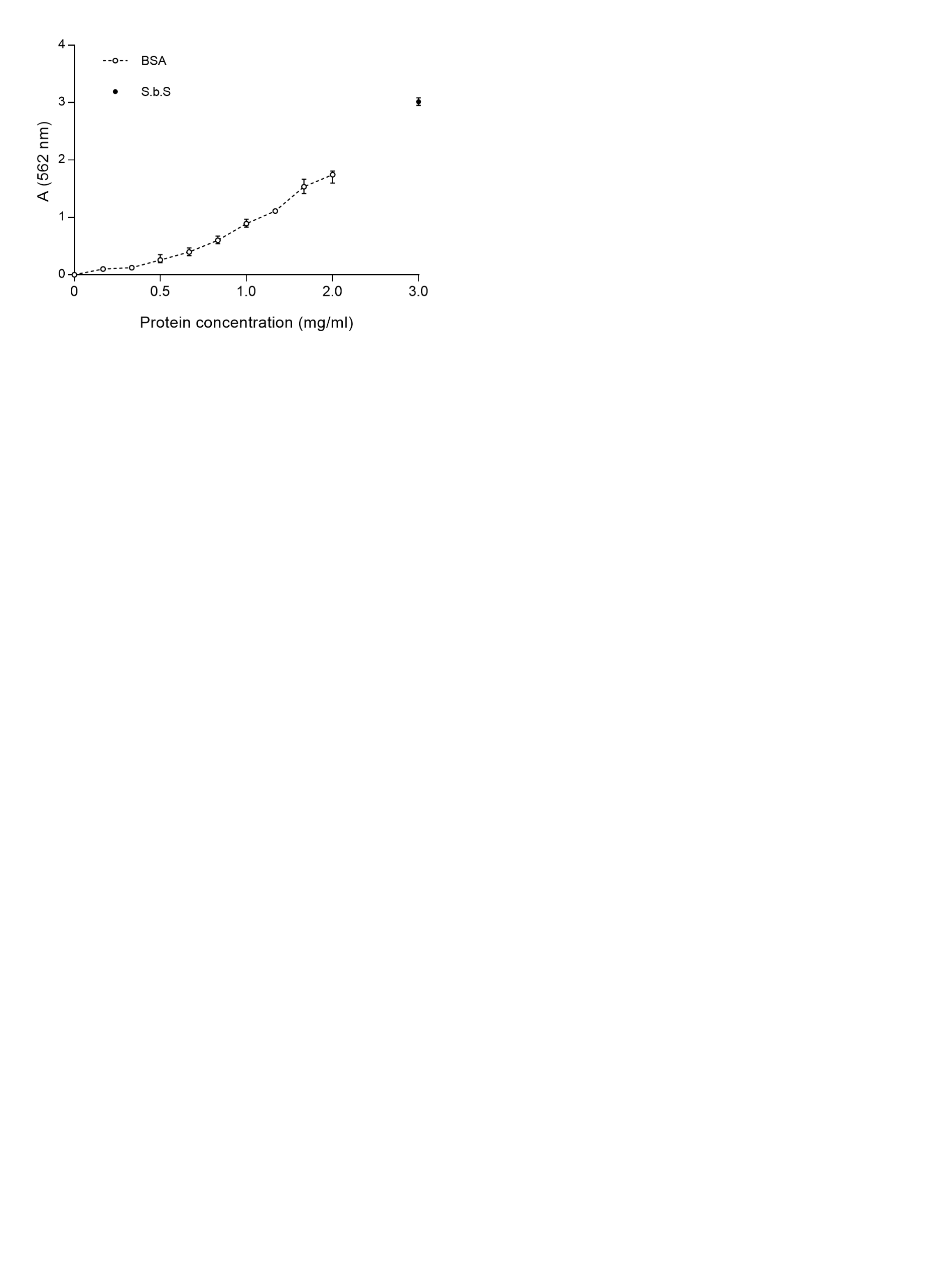


Figure S1. Determination of protein concentration in S.b.S by BCA protein assay. 10-point standard curve created by BSA dilutions. For each standard dilution triple absorbance, measured by fluorometric, was averaged and plotted on the y-axis. Data are presented as means ± SEM.

Figure S2:


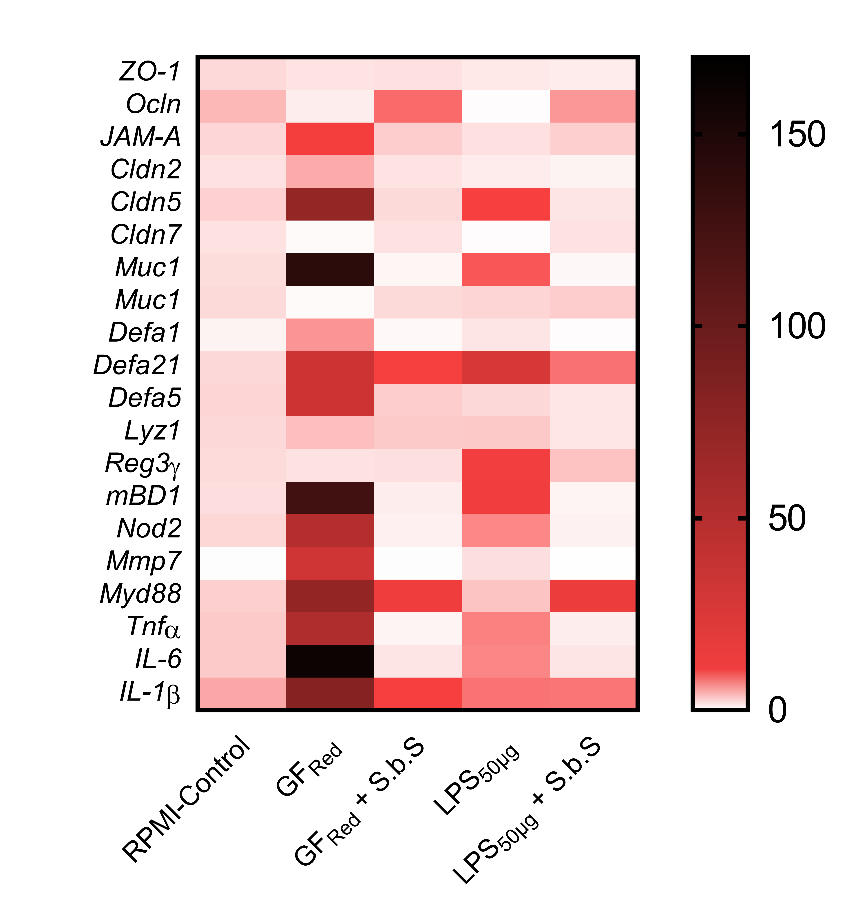


Figure S2. Heatmap of relative gene expression levels of tight junction proteins, mucins, antimicrobial peptides, and inflammatory markers in murine small intestinal organoids. Murine small intestinal organoids were treated with S.b.S (200 µg/mL), or GF_Red_ ± S.b.S (200 µg/ml), or LPS (50 µg/mL) ± S.b.S (200 µg/mL) or RPMI as control for 30 h at 37°C. Gene expression was measured by RT-PCR and normalized to β-actin. Data are presented as means ± SEM (n = 8). Color scale indicates increasing expression from white (0%) to dark red (up to 170%).

Figure S3


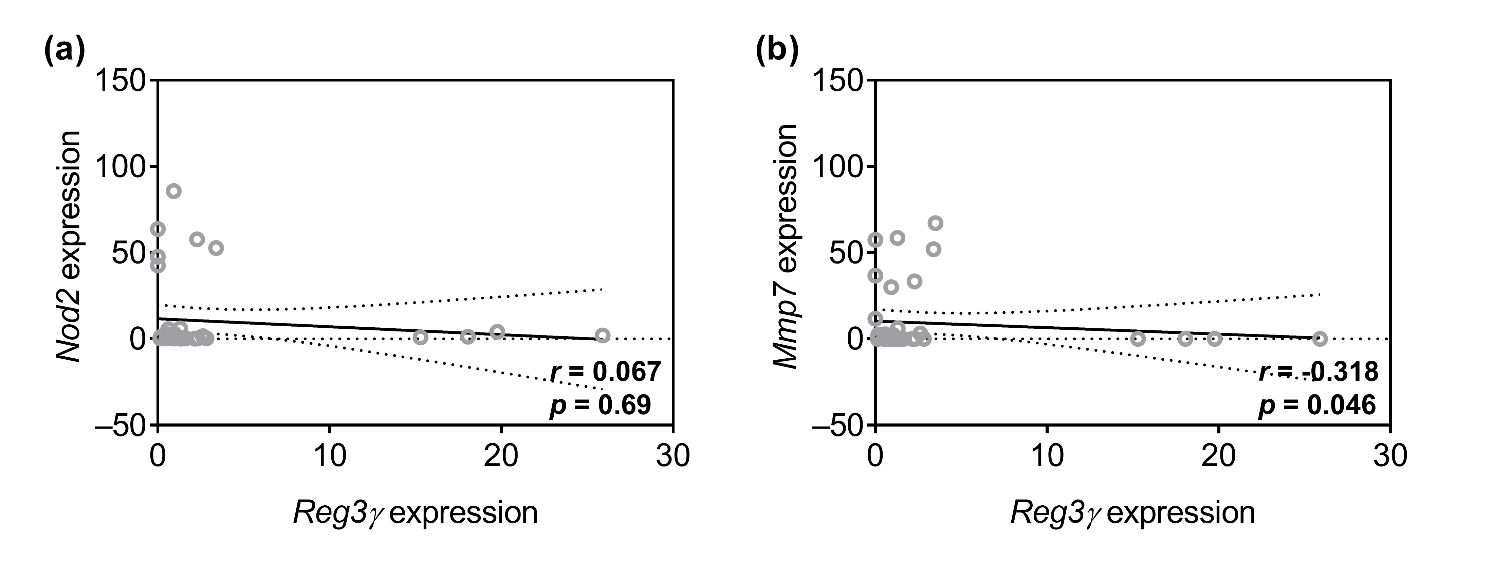


Figure S3. Correlation analysis for *Nod2* **(a)** or *Mmp7* **(b)** and *Reg3γ* mRNA expression. Statistical analysis was performed by two-tailed Spearman rank correlation analysis. Correlations were defined as: 0.0 to 0.2 no correlation; -0.2 to -0.4, weak negative correlations.
